# Exploring larger evidence-base for contemporary Ayurveda

**DOI:** 10.4103/0974-7788.64394

**Published:** 2010

**Authors:** Ram Harsh Singh

**Affiliations:** *Professor Emeritus, Faculty of Ayurveda, Banaras Hindu University, Varanasi, India E-mail: rh_singh2001@yahoo.com*

Ayurveda represents the most ancient and classical knowledge base pertaining to life science, health and cure, its antiquity going back to the *Vedas*. It seems to have been the world view of its time, although subsequently the world view of this knowledge base shrank to India alone and India remained its sole custodian till the end of the 20^th^ century. Because of its unique pro-nature vision, Ayurveda once again is gaining global relevance. This new upsurge of interest in Ayurveda and its rapidly increasing public use has given rise to many newer issues and challenges. The public in general, as well as the scientific and professional community, seems to be largely convinced with the rationality and possible scientific validity of the principles and approaches of Ayurveda as a logical life science and as a healing modality. But simultaneously the demand for new scientific evidence for the efficacy, safety and quality of its medications is gaining momentum. Such a demand has motivated a large number of investigators to launch ambitious research and development activities. This is definitely a welcome development.

However, it cannot be overemphasized that ongoing research and development activities do not seem to be based on sound footings. Most of the current R and D programs in this field are still based on the conventional reductionist methodology, which is often applied by molesting the holistic theories of Ayurveda. There is a need to develop new appropriate research methodology for Ayurvedic research through intense interface between Ayurveda and conventional science. It is because of lack of such an appropriate research methodology that Ayurvedic research has not succeeded in yielding any breakthrough in recent years. It cannot be overemphasized that in any research exercise the goal of research should not be compromised to suit the convenience of researching methods.

The overall spectrum of contemporary Ayurvedic research seems to include (1) Literary and conceptual research; (2) Clinical and therapeutic research; (3) Drug development research, including standardization of in-use drugs and development of new drugs. In my perception, the only successful component of Ayurvedic research activity during the last five decades has been the literary research conducted by scholars of Ayurveda, including the scholarly works and translations and critical commentaries on Ayurvedic classics in modern languages which made the classical knowledge base accessible to the present generation of scholars and scientists, thereby opening newer vistas of research and development.

As a matter of fact, now Ayurveda requires two-pronged research enterprise, namely, (1) Research in the science of Ayurveda and (2) Research in the therapeutics of Ayurveda. Till now, the entire effort seems to have been focused on therapeutic research, that too in a halfhearted manner, borrowing abridged methodology without any genuine attempt to develop appropriate new methods specific to the Ayurvedic approach. As a result, the research so done has not proved really rewarding. The former sector, i.e., the research in science of Ayurveda, has remained largely unexplored, although now there is a gradual paradigm shift, which can be visualized in Dr. Valiathan's project on *Science Initiative in Ayurveda*.

On the other hand, one should not go with the impression that classical Ayurveda has no evidence base. In fact, Ayurveda has always been evidence conscious, and most of the principles and treatment modalities seem to have been critically tested and validated for the conditions existing in their own time frame. The ancient concept of evidence is based on fourfold testing, viz., (1) *Pratyaksha pramana* (direct observation), (2) *Anumana pramana* (inferential evidence), (3) *Aptopadesa* (scriptural evidence) and (4) *Yukti pramana* (planned rational experimental evidence). This fourfold battery of testing new knowledge is classical of ancient Indian scientific tradition, which seems to be highly contemporary.

The evidence base of contemporary Ayurveda is to be visualized in several forms, including (1) Textual evidence and folklore claims, (2) Experience-based evidence, (3) Longstanding traditional use, (4) Mass acceptability and (5) New scientific evidence. It cannot be overemphasized that in spite of all the strengths of primary evidence, one cannot deny the need to develop new supportive scientific evidence without which contemporary Ayurveda cannot attain the status of a real global science accessible for the larger benefit of humanity at large. WHO also holds a similar view. However, it must be emphasized that fruitful strategies for developing new scientific evidence cannot succeed if traditional primary evidence is ignored. New research is to be planned on the foundations of existing textual and experience-based evidence. The frequently used term "evidence" essentially means a relevant and reasonable proof for a fact or truth; such a proof need not be necessarily in words or terms of today's science alone.

The technology for developing new scientific evidence for the validity of a classical fact or concept will have to be created afresh precisely to test the original knowledge without distorting the same to suit the application of the new methodology. Any undue compromise in this regard is unwarranted because Ayurvedic biology and therapeutics are fundamentally different than the contemporary sciences, hence routine modern research methods will prove futile. I personally feel that at present there is more a need of research in developing an appropriate research methodology for Ayurvedic research than actual clinical or therapeutic research in Ayurveda.

I would like to illustrate the point through an example of research in *panchakarma* therapy. *Panchakarma* is the therapeutic technology of *samsodhana*, while *samsodhana* is the fundamental therapeutic principle of Ayurveda. According to Ayurveda, the living human body is comprised of innumerable channels, i.e., *srotamsi* — "*srotomayam hi sariram*," which functions as a quantized inner transport system of the living body, both gross and subtle, biologic and energetic, as well as tangible and intangible. Because of the ongoing life processes, pollution and breakdown, the *srotamsi* are inherently prone to lose their integrity and need to be periodically cleansed with the help of appropriate therapeutic technology such as *panchakarma*. Any research in *panchakarma* therapy and *samsodhana* in contemporary perspective needs to develop an appropriate research methodology which may directly throw light on the integrity of *srotas* function. I wonder if any such study could be done at the level of membrane biology as known in contemporary life science. Whether a *samsodhana* treatment really improves the integrity of membrane function at the visceral and cellular level will be of real interest. This is important in view of the fact that most of the chronic diseases, including inflammatory and immunological diseases, involve membrane pathology leading to cellular and visceral failure; and in all such cases, *samsodhana karma* may prove crucial.

Similarly *rasayana* therapy of Ayurveda is primarily linked with molecular nutrition. Ayurvedic *rasayana* remedies, including *achara rasayana, ajasrika rasayana* and *ausadhi rasayana*, seem to work through nutrition dynamics, improving the molecular health (*dhatu samya*), leading in turn to rejuvenation and positive health (*swasthya*). In view of such facts, a research programme in *rasayana therapy* should focus on mechanism studies before proceeding to parametric assessment of the secondary effects, which could be the mixed effect of many associated factors. It cannot be overemphasized that only basic studies may succeed to validate the basic principles of Ayurveda, which would open newer vistas of original research. The secondary therapeutic studies will limit the scope to mere treatment validation, with temporary impact.

On similar lines, urgent action is needed to study the unique concepts of *tanmatra, tridosa, saptadhatu, ojas, ojabala, baladosa, agni, agnibala, ama, srotas*, etc., besides the concept of *rasa, guna, virya, vipaka, prabhava* of drugs, which projects unique holistic pharmacology and pharmacodynamics of the drugs used in Ayurveda. Such attempts are likely to help developing a new positive approach to drug research, introducing new traits of medications and revolutionizing the entire field of medicine and medical care.

